# Multivariate time series dataset for predicting surface roughness and dimensional error in longitudinal turning

**DOI:** 10.1016/j.dib.2026.113325

**Published:** 2026-10-09

**Authors:** Berend Denkena, Benjamin Bergmann, Henning Buhl, Dennis Kowalke, Miriam Handrup

**Affiliations:** Institute of Production Engineering and Machine Tools, Leibniz Universität Hannover, 30823 Garbsen, Germany

**Keywords:** Turning, Time series, Surface roughness, Diameter error, Predictive quality

## Abstract

Quality control in machining is essential for consistent workpiece quality. Predictive quality methods that predict the workpiece quality based on monitored signals are used instead of real measuring devices in order to reduce non-productive times and save costs by detecting scraps already during the process. The methods often apply machine learning models since they can model complex relationships and can handle a wide range of signal sources. While machine learning models require a lot of training data, only few datasets are publicly available that include process signals from various sensors. The presented dataset provides multivariate time series data of 453 longitudinal turning processes with varying feed, cutting speed, depth of cut and workpiece material. Additionally, the resulting surface roughness and diameter deviation are provided for each cut. All cuts were carried out using new cutting tools. To minimize measuring errors, the surface roughness and diameter deviation were measured on-machine using machine integrated touch probes. This dataset supports research into predictive quality using machine learning models.

Specifications Table

**Table:** 

Subject	Engineering & Materials science
Specific subject area	Surface roughness and dimensional error prediction in longitudinal turning
Type of data	Filtered multivariate time series including metadata
Data collection	Turning experiments were conducted on a machine tool. The process forces were recorded with a dynamometer, and the tool acceleration was measured with an acceleration sensor. Additionally, the torque of the drives and the spindle, the axis positions, and the position control deviations were recorded from the machine tools’ control. For each machined surface the resulting diameter and the surface roughness were measured using on-machine touch probes.
Data source location	Recorded and stored at the Institute of Production Engineering and Machine Tools, Leibniz Universität Hannover, 30,823 Garbsen, Germany
Data accessibility	Repository name: Research Data Repository of the Leibniz Universität Hannover Data identification number: https://doi.org/10.25835/9xffqwnc Direct URL to data: https://data.uni-hannover.de/dataset/multivariate-time-series-dataset-for-predicting-surface-roughness-and-dimensional-errors-in-turning
Related research article	None

## 1 Value of the Data

• The data can be used to develop machine learning models for predicting surface roughness and dimensional errors in longitudinal turning processes based on multi-sensor process signals. Multi-sensor data availability enables identification of the most useful sensors, while minimizing the number of sensors and their corresponding cost for practical application.
• The data is valuable as it includes various feeds, cutting speeds and depths of cut, as well as three different workpiece materials.
• The data provided in this publication adds to the currently very limited amount of publicly available time series data for machining. It benefits researchers and product developers, as it reduces the need to conduct additional experiments.
• In addition to developing quality prediction models, the dataset can be used for other applications such as the prediction of process forces with machine internal process signals.

## 2 Background

The data was recorded to develop a machine learning model for predicting workpiece quality features, especially the resulting surface roughness and dimensional error. In the Collaborative Research Centre 1153, the production of multi-material workpieces is the subject of ongoing research [1]. The idea is to use costly high-alloyed materials only in areas of the component where necessary. However, the use of different materials in a workpiece leads to challenges in machining. Especially, the resulting dimensional error and the surface roughness are being influenced by the workpiece material. To enable the control of the final workpiece quality by adapting the process parameters, a model is required that can predict the resulting workpiece quality for varying materials and process parameters. Previous research on surface roughness prediction for multiple materials, typically using smaller datasets, like [2], can be used as benchmark models.

## 3 Data Description

All experimental data is saved in one dataset called “Database.h5” file (HDF5 format). The structure of the .h5 file is depicted in Fig. 1. In the dataset, there are three sample groups, each representing a different sample material. These three sample groups are named after the unique sample ID of each probe, namely K0025, K0026 and K0027. To describe the probes, attributes are added to each sample group. These attributes include, for example, the sample material and hardness. An overview of all provided attributes can be found in Table 1, and the dataset is available at [3].

**Fig. 1:**
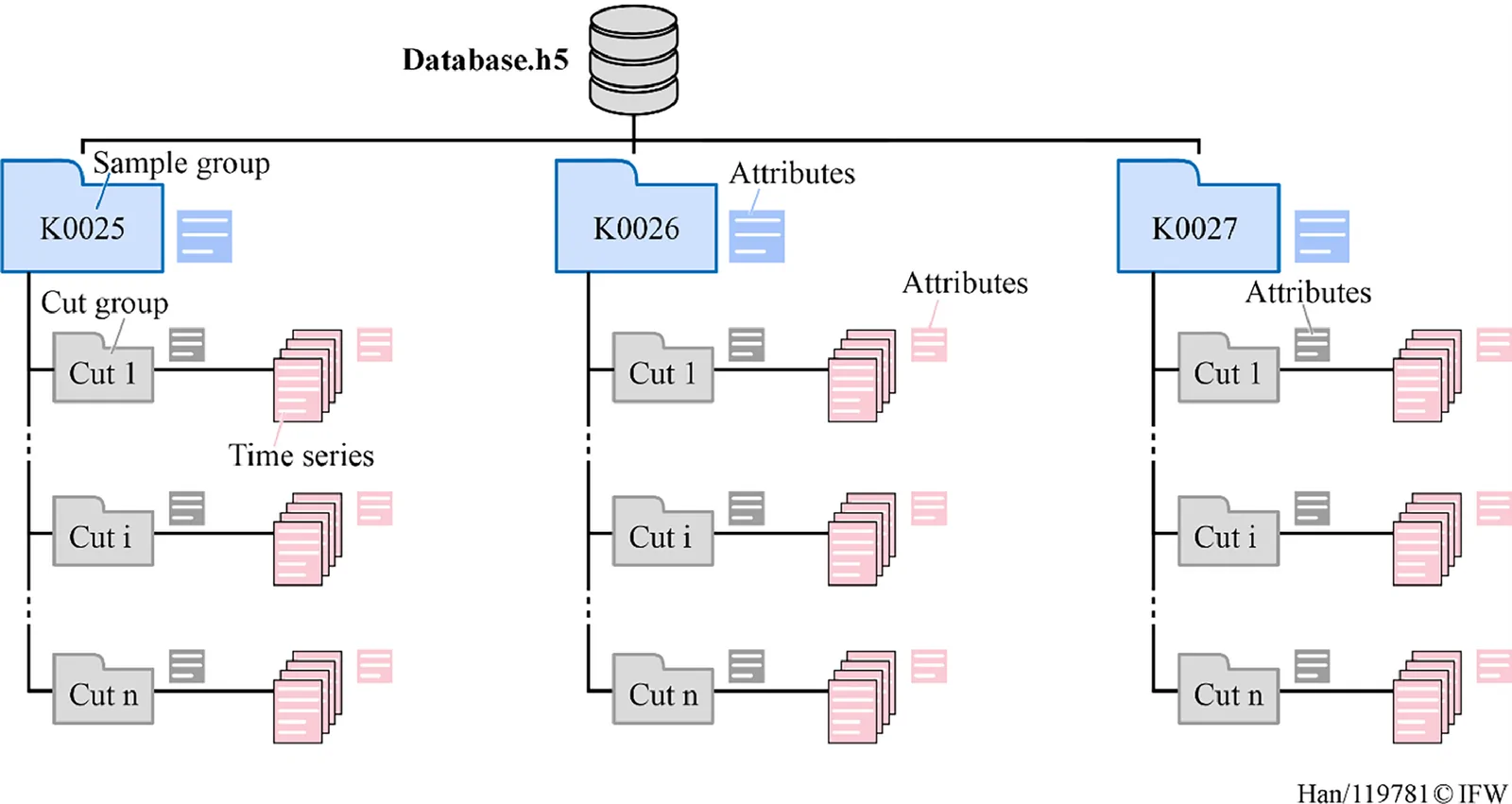
HDF5 database structure.

**Table 1:** Description of attributes in “Database.h5” file.

Group	Name	Description	Unit	Format
Sample	Batch Number	Delivery date of the charge	-	String
	Description	Description of the process	-	String
	HV	Vickers Hardness of sample material	HV1	Float64
	SampleMaterial	Material description	-	String
	Tool	Tool description	-	String
	Unique Sample Id	Sample identification	-	String
Cut	Measurement_ID	Identification number of the measurement	-	Int64
	Ra	Surface roughness Ra	µm	Float64
	Rz	Surface roughness Rz	µm	Float64
	T_error	Tool length error T_err_	mm	Float64
	T_length	Tool length in the X-axis direction	mm	Float64
	ap	Depth of cut a_p_	mm	Float64
	d0	Previous diameter d_0_ (measured)	mm	Float64
	d0_nominal	Previous nominal diameter d_0,nominal_	mm	Float64
	d1	Machined diameter d_1_ (measured)	mm	Float64
	d1_nominal	Machined nominal diameter d_1,nominal_	mm	Float64
	d_error	Dimensional error d_err_	µm	Float64
	f	Feed f	mm	Float64
	num_segments	Number of segments in measurement	-	Float64
	re	Corner radius r_ε_	mm	Float64
	vc	Cutting speed v_c_	m/min	Float64
Time series	sampling_rate	Sampling rate f	Hz	Float64
	segment_start	Segment start time	s	Float64
	segment_stop	Segment stop time	s	Float64
	signal_source	Sensor source of the signal	-	String

In each sample group, there are multiple cut groups which each represent the data of a single cut. In total, the dataset includes 453 longitudinal turning cuts with a length of 10 mm. The cut groups are named based on their recording date in the date format “Scope_yyyy_MM_dd_HH_mm_ss”. Cut-specific attributes, such as the process parameters or the corner radius of the tool, are defined as attributes of the cut groups. In addition, the workpiece quality containing the resulting diameter and surface roughness for each cut is also saved as attribute.

In each cut group, the process signals are stored as an equidistant time series. To reduce the required memory space, no time vector is given in the time series, but instead, the sampling rate of each signal is defined as an attribute so that the time vector can be easily reconstructed if required. The recorded process signals are listed in Table 1, either recorded from the machine tool numerical control unit, an acceleration sensor or a dynamometer. An example of the various time series recorded is given in Fig. 2. The axis positions are given in the workpiece coordinate system (WCS). The X- and Z-axes refer to the axes of the turret. The corresponding coordinate system is depicted in Fig. 3. The signals were additionally segmented to isolate constant cutting conditions. Each segment is exclusively limited by the position of the Z-axis within the range of -2 mm to -7 mm. This area was chosen to match the area of tactile probe measurements. The calculated segment start and stop time are provided as an attribute for each time series. Thus, significant signal features can be generated by evaluating the signals in the defined time range.

**Fig. 2:**
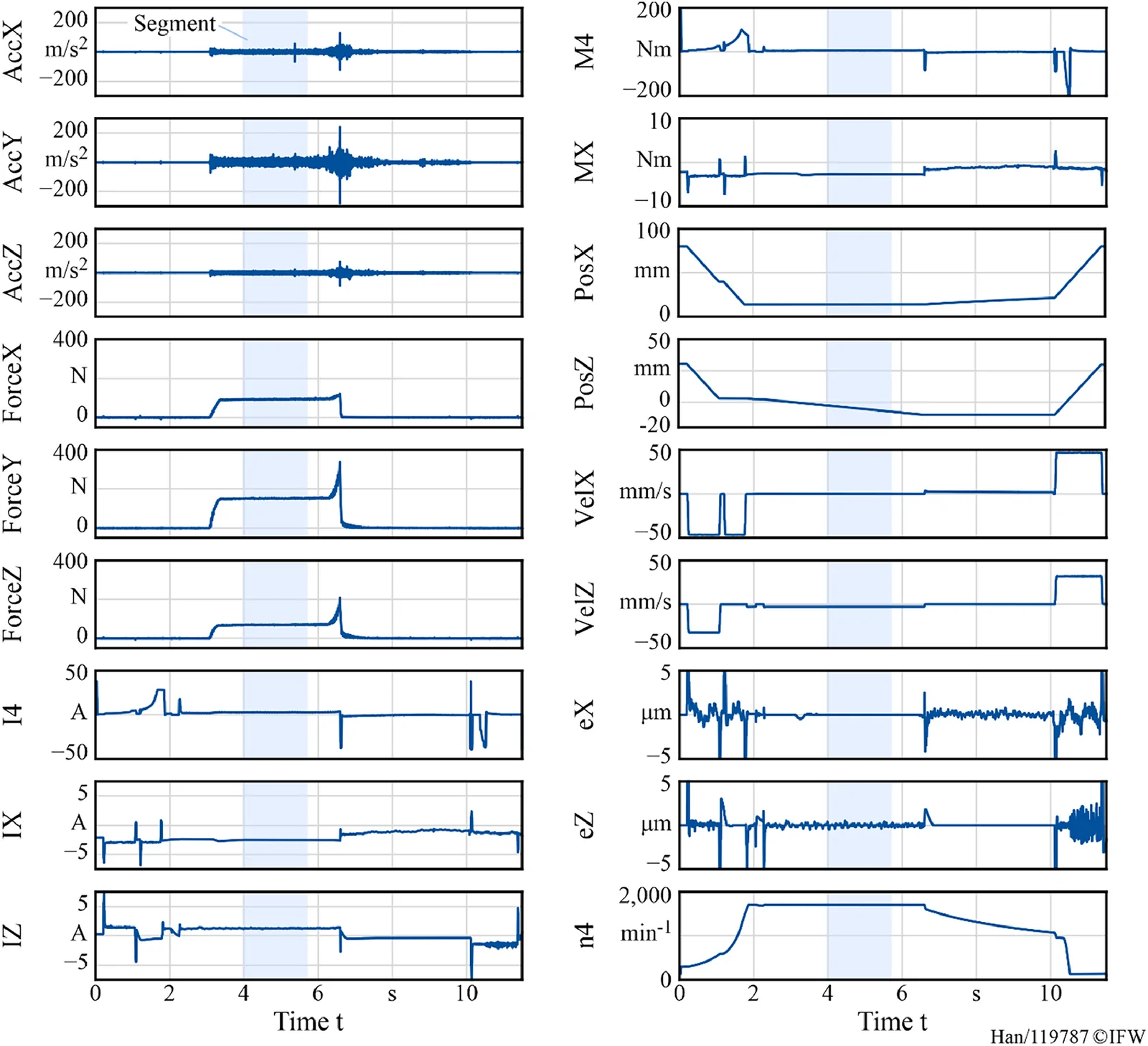
Time series data recorded of an exemplary cut.

**Fig. 3:**
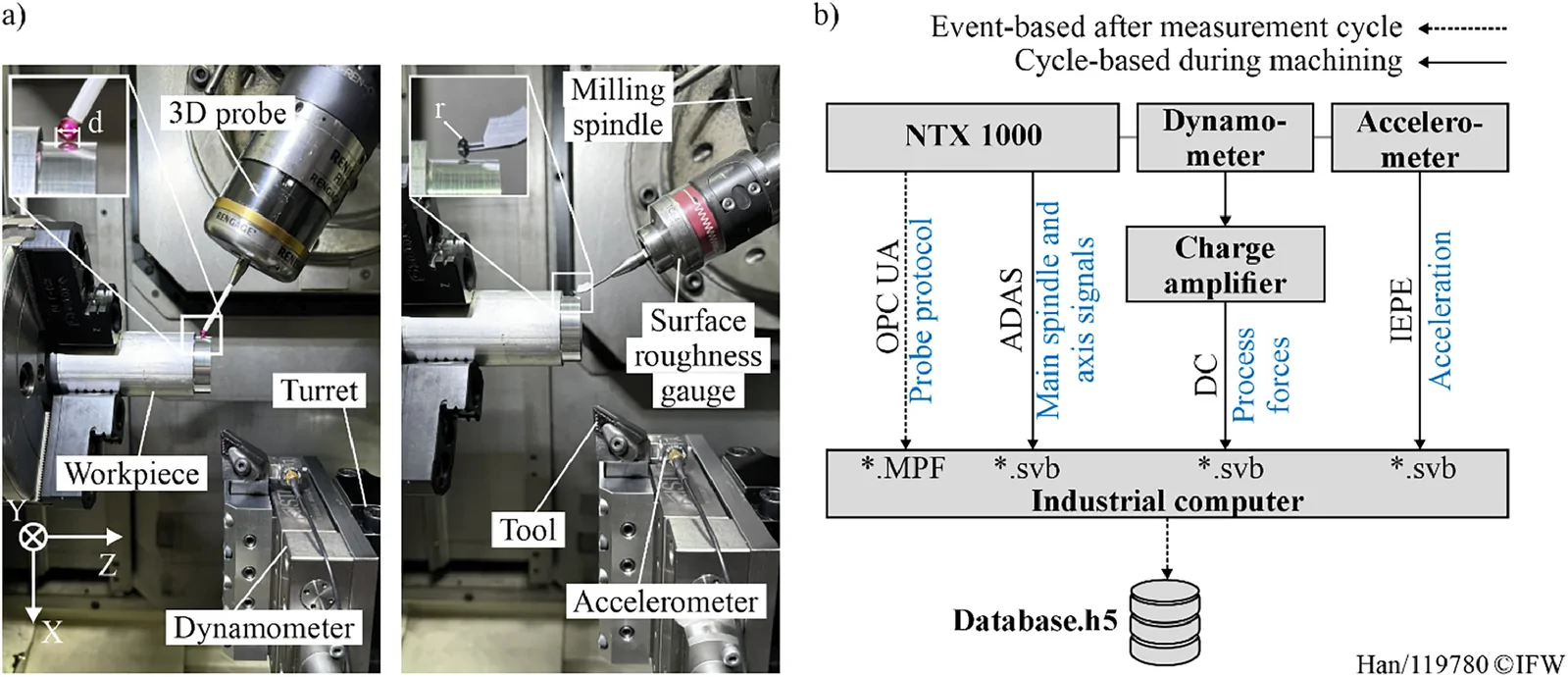
Experimental setup and data acquisition setup.

## 4 Experimental Design, Materials and Methods

The goal of the experiments was to acquire process data, the resulting surface roughness, and dimensional error for various process parameters and workpiece materials. The measurements were recorded in longitudinal dry turning of cylindrical probes. For machining a DMG Mori NTX 1000 mill-turn center with Siemens Sinumerik 840D sl control was used with a milling spindle above the main spindle and a turret below. In the milling spindle, the touch probes for on-machine workpiece quality measurement can be mounted from the magazine of the machine tool. Both setups are shown in Fig. 3a. The tool was mounted on a Kistler 9129AA dynamometer which was mounted in the turret. Thus, the dynamometer was directly in the force flow. For the dynamometer, charge amplifier 5015A from Kistler were used with an integrated low-pass filter of 100 Hz. Furthermore, a triaxial accelerometer KS903B from MMF with a sensitivity of 100 mV/g and a measuring range of ±60 g was mounted to the cutting tool. In addition to the sensor signals, various machine internal signals were extracted from the numerical control unit of the machine tool using the ADAS (“Axis Data Stream”) compile cycle. The ADAS compile cycle updated the selected signals every 10 ms. All signals were recorded with a Beckhoff industrial computer using various analog input modules. The measured signals were saved in a separate .svb file. The different signal sources are responsible for the different sampling rates of the signals, that are given in Table 2. The data acquisition setup is depicted in Fig. 3b The recording was automatically started and stopped at the beginning and the end of the rotation of the main spindle. Accordingly to the low-pass filter within the charge amplifier and the ADAS cycle time, the data acquisition for non-acceleration signals was limited to 1 kHz. The acceleration data was recorded with the maximum frequency of the used IEPE input modules at 20 kHz.

**Table 2:** Description of time series data in “Database.h5” file.

Signalname	Description	Source	Unit	Sampling rate f	Format
AccX	Acceleration of the tool in the X-axis direction	Sensor	m/s^2^	20 kHz	Float32
AccY	Acceleration of the tool in the Y-axis direction	Sensor	m/s^2^	20 kHz	Float32
AccZ	Acceleration of the tool in the Z-axis direction	Sensor	m/s^2^	20 kHz	Float32
ForceX	Force in the X-axis direction	Sensor	N	1 kHz	Float32
ForceY	Force in the Y-axis direction	Sensor	N	1 kHz	Float32
ForceZ	Force in the Z-axis direction	Sensor	N	1 kHz	Float32
I4	Actual main spindle current	Machine	A	1 kHz	Float32
IX	X-axis drive actual current	Machine	A	1 kHz	Float32
IZ	Z-axis drive actual current	Machine	A	1 kHz	Float32
M4	Main spindle torque setpoint	Machine	Nm	1 kHz	Float32
MX	X-axis drive torque setpoint	Machine	Nm	1 kHz	Float32
PosX	Actual position of the X-axis drive in WCS	Machine	mm	1 kHz	Float32
PosZ	Actual position of the Z-axis drive in WCS	Machine	mm	1 kHz	Float32
VelX	Actual velocity of the X-axis	Machine	mm/s	1 kHz	Float32
VelZ	Actual velocity of the Z-axis	Machine	mm/s	1 kHz	Float32
eX	Actual position error of the X-axis	Machine	µm	1 kHz	Float32
eZ	Actual position error of the Z-axis	Machine	µm	1 kHz	Float32
n4	Main spindle speed	Machine	min^-1^	1 kHz	Float32

The workpieces were shafts with a raw diameter of 40 mm consisting of 20MnCr5, EN AW-6082 T6 or 41Cr4, as they are exemplary materials used in current research on production of multi-material workpieces. For machining, new Kennametal DNMG150608 KCS10B inserts with a corner radius of 0.8 mm and a Sandvik DDJNL 2020 K 15 tool holder were used. A tool cutting edge angle of κ = 93° and a clearance angle of α = 5° were used. The tool inserts used for the cutting experiments are suited for machining stainless steel and high-temperature steel alloys. Therefore, the tools are well suited for cutting 20MnCr4 and 41Cr4. The tools are not well suited for cutting the aluminum alloy. Because continuous cutting is required to finish multi-material workpieces, changing the cutting insert at the often unknown material transition is not suitable. Therefore, the same type of cutting insert is used for all three materials. The Vickers hardness (HV1) and chemical composition of each material are listed in Table 3. In the main spindle chuck, the workpiece was clamped with a cantilever length of 20 mm for a 10 mm long cut, so that the effect of workpiece deflection is minimal while ensuring clearance between the cutting tool and the chuck. To ensure a specific depth of cut in all experiments and eliminate fixture-related effects on the diameter error, the shafts were pre-turned to a diameter of 39 mm with cutting speed v_c_ = 200 m/min, feed f = 0.15 mm/rev and depth of cut a_p_ = 0.5 mm. These cuts were also used for referencing the tool length error by comparing the measured reference diameter d_reference_ with the nominal diameter d_nominal_ = 39 mm. The tool length error T_error_ was only calculated for the referencing cut and is assumed to be constant for the subsequent cuts:

(1) $$T_{error}=\frac{\left(d_{nominal}-d_{reference}\right)}{2}$$

**Table 3:** Material description.

Material	Material number	Hardness in HV1	Chemical composition by Optical Emission Spectroscopy (OES) Concentration in %
20MnCr5	1.7147	175	Fe 96.84 - Mn 1.1800 - Cr 1.1000 - Si 0.2820 - Cu 0.2150 - C 0.1630 - Ni 0.0802 - Al 0.0234 - S 0.0201 - Mo 0.0168 - other 0.0795
EN AW-6082 T6	3.2315	102	Al 97.52 - Si 0.8830 - Mg 0.7170 - Mn 0.4420 - Fe 0.2600 - Cu 0.0392 - Zn 0.0350 - Ti 0.0309 - Cr 0.0252 - other 0.0477
41Cr4 + QT	1.7035	300	Fe 97.09 - Cr 1.0200 - Mn 0.7760 - C 0.3760 - Si 0.3410 - Cu 0.1400 - Ni 0.1150 - Mo 0.0269 - S 0.0203 – other 0.0948

It should be noted, that the tool length error includes all effects that lead to a dimensional error in the referencing cut. These are mainly the calibration errors of the tool length, but can also include other effects such as tool deflection or elastic workpiece material behavior during cutting. The tool error is stated in the data set but not used to compensate the nominal and measured diameters. The workpiece material-specific influences of the referencing cut are compensated by applying a constant offset, which was previously measured by machining referencing cuts on all workpiece materials without changing the cutting edge. For the calculation of the material-specific offset, the diameter error of 20MnCr5 is the baseline.

After the referencing cut, the workpieces are machined using various cutting speeds, feeds and depth of cut until a minimum workpiece diameter of 19 mm is reached. After the workpiece diameter reached 19 mm, the remaining material in that area is removed, and the next workpiece area of 10 mm length is pre-turned, the tool length is again referenced, and the experiments continue. The process parameters, arranged at specific grid points, are shown in Fig. 4b In addition to the marked parameters, 75 cuts at random parameters within the grid of 0.5 mm ≤ a_p_ ≤ 1 mm, 100 m/min ≤ v_c_ ≤ 300 m/min, and 0.1 mm ≤ f ≤ 0.3 mm were performed. These cuts are distributed equally among the three workpiece materials and are intended for testing the trained prediction model. As a result of the regularly distributed grid points and the random parameters, the interpolation capability and possible overfitting of the model can be effectively evaluated. The edges of the grid were identified using preliminary cutting experiments on all three materials. The experimental sequence within each material was not carried out in the same sequence. The sequence can be obtained from the name format of each cut.

**Fig. 4:**
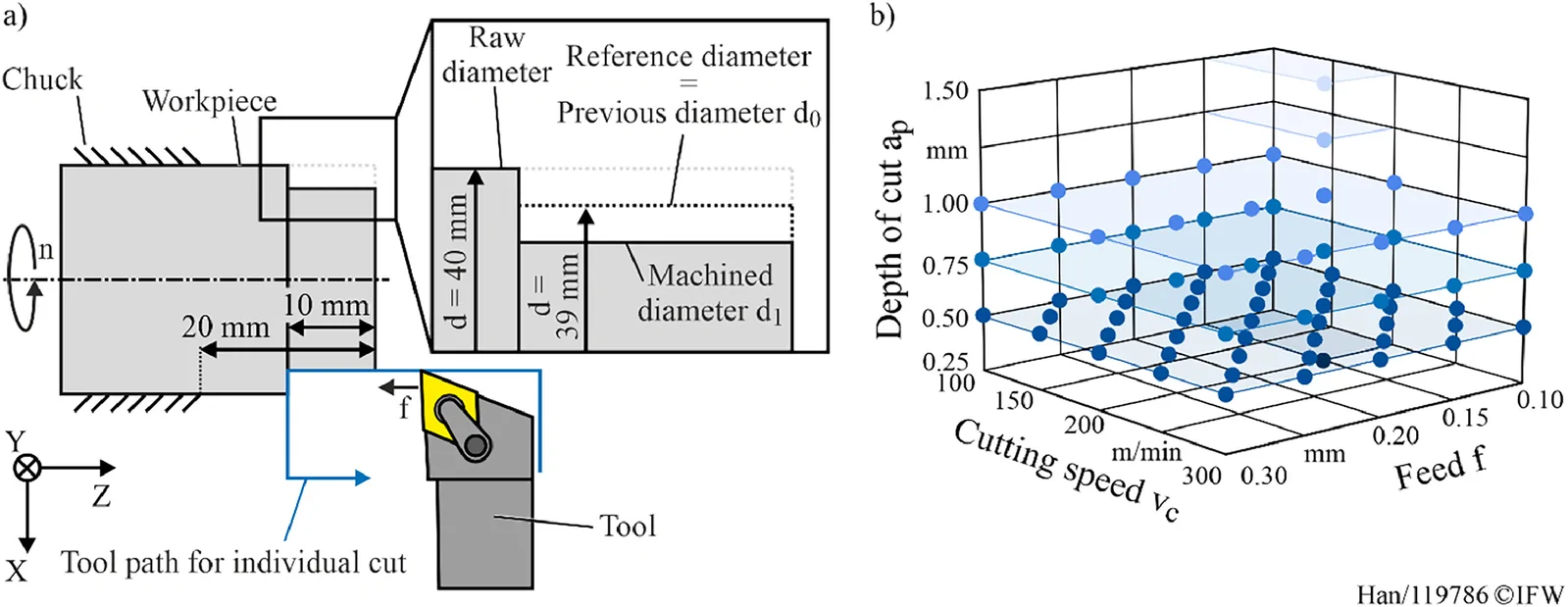
a) Process design, b) Process parameters.

After each cut, the machined workpiece quality was measured using on-machine touch probes. For the diameter measurement, a Renishaw RMP600 with a ball diameter of d = 4 mm was used. It has a 3D-measuring uncertainty of ±1 µm (2σ). Here, the measuring speed was set to 100 mm/min. Two opposite points on the workpiece were probed, and the results were averaged. At the final workpiece diameter of 19 mm, a maximum deflection of <150 nm is expected in accordance with the theoretical Deflection of a Cantilever Beam for aluminum and a process force of 200 N. Therefore, the variation in deflection with decreased diameter is well below the measument accuracy of the touch probe. This also supports the hypothesis that the tool error can be assumed constant. For surface roughness measurement, a Blum TC64-RG with a tip radius of r = 20 µm was used. The measuring speed was set to 50 mm/min and the cut-off wavelength to λ_s_ = 0.8 mm. The accuracy of the roughness gauge has been experimentally evaluated by machining 55 different surface roughnesses and comparing the result to the surface roughness measured with a Mahr MarSurf LD 130. The results are shown in Fig. 5a. A systematic error was found, as the on-machine gauge undervalues the actual roughness. This systematic error could be caused by mounting errors, misalignment of the sensor in the machine or a lower resolution compared to the roughness measuring station in the lab. The systematic error was evaluated and compensated with a static offset for all measurements, which is shown in Fig. 5b for the surface roughness Rz. The static offset is calculated based on the mean error. The offset for Rz is 0.944 µm. The error of the on-machine measurement after compensation is ±1.38 µm (3σ) for Rz and is therefore considered reliable. For the surface roughness Ra, the same procedure was applied. The offset is 0.1508 µm and the resulting error is ±0.36 µm (3σ). The data set contains the compensated surface errors for Ra and Rz.

**Fig. 5:**
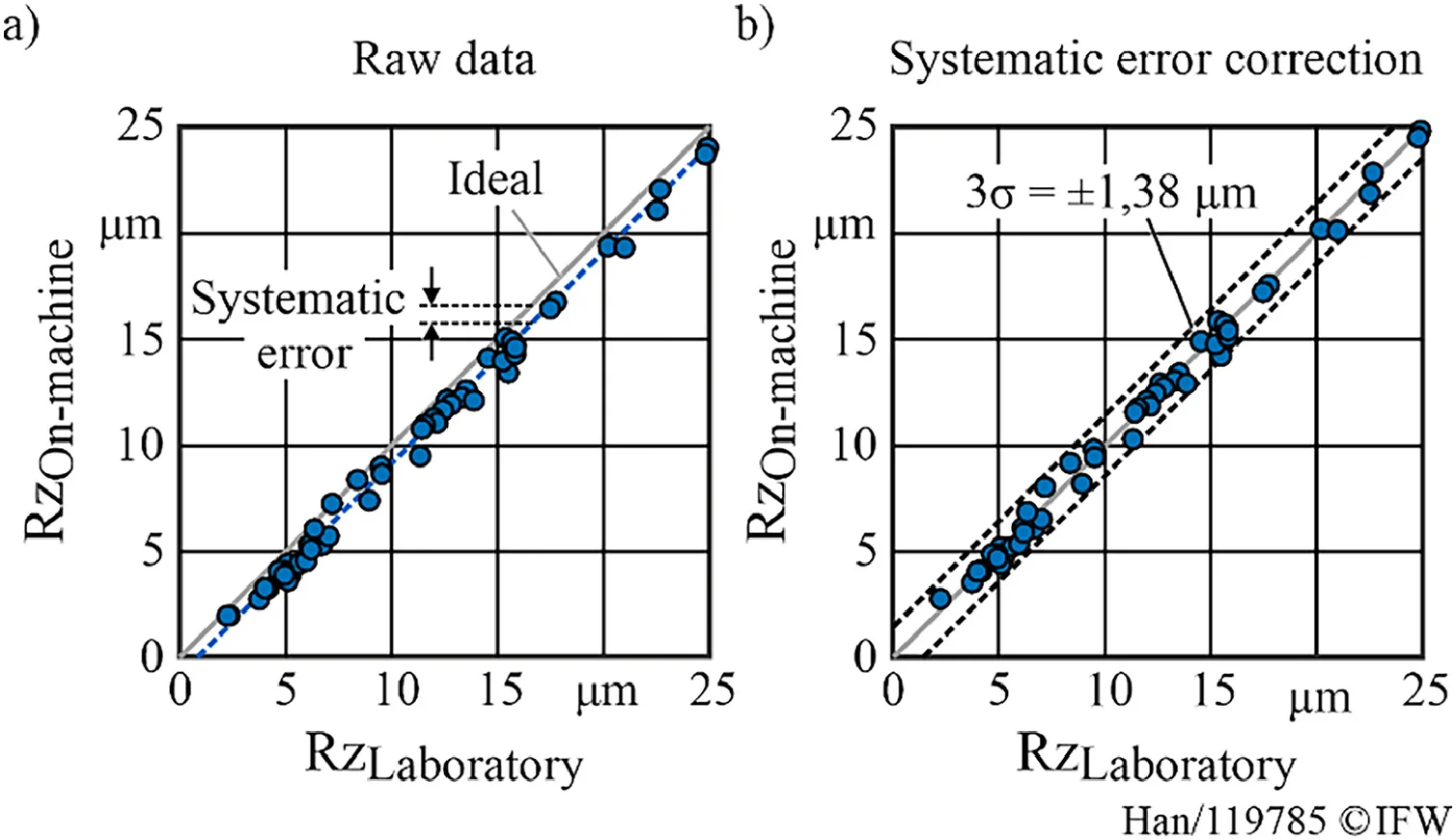
a) Raw data, b) Systematic error correction of the surface roughness measurement.

The numerical control of the machine tool saved the measured diameter d_1_ and the surface roughness variables Ra and Rz at the end of the measurement in a .MPF probe protocol file. After finishing the experiments, the time series and the probe results were saved in the “Database.h5” file described previously. The dimensional diameter error d_err_ was calculated by comparing the measured diameter d_1_ to the nominal diameter d_1,nominal_ subtracted with the constant tool length error T_error_:

(2) $$d_{err}=d_{1}-\left(d_{1,nominal}-\;2\cdot T_{err}\right)$$

The referencing process of the tool length error was carried out for every single machined 10 mm area of each material. The measured dimensional error is therefore relative to the previous referencing.

## Limitations

In some cuts, the acceleration signal got into overflow due to chips hitting the acceleration sensor. Most overflow effects are minimal and are removed by the high pass filtering of the IEPE input modules. However, in some cases greater peaks remain, as shown e. g. in K0027/ Scope_2025_06_26_16_46_21 and K0026/Scope_2025_11_13_10_13_57. As chips hitting the acceleration sensor is a practical problem in practical machining and difficult to observe, the data is not further annotated.

Effects while cutting aluminum with the used tool inserts can include built-up-edges and adhesion on the cutting edge. Those effects directly influence the surface roughness, the diameter error and the process signals. These and other effects within the cutting process were not monitored beyond the process signals and are therefore not included in the dataset.

The usage of new cutting tools excludes the influences of tool wear on surface roughness and dimensional error. The tool wear also influences the cutting forces, acceleration and the current of the machine axis. Therefore, for the application of data driven models in practical machining this needs to be considered. Due to the data export between each cut and overall low chip removal, thermal drift is expected to have no influence. For applications in practical machining, this factor needs to be considered.

## Ethics Statement

The data provided is original and is free of secondary data or data collected from social media platforms. Data collection did not involve any human subjects, animal experiments, chemicals, procedures or equipment that have any unusual hazards inherent in their use.

## Credit Author Statement

**Berend Denkena**: Supervision; **Benjamin Bergmann**: Supervision; **Henning Buhl**: Writing – review & editing; **Dennis Kowalke**: Writing – review & editing; **Miriam Handrup**: Conceptualization, Methodology, Software, Formal analysis, Data curation, Writing – original draft.
